# Bionomic aspects of the *Anopheles subpictus* species complex in Sri Lanka

**DOI:** 10.1093/jis/14.1.97

**Published:** 2014-07-22

**Authors:** Pavillupillai J. Jude, Ranjan Ramasamy, Sinnathamby N. Surendran

**Affiliations:** 1 Department of Zoology, Faculty of Science, University of Jaffna, Jaffna, Sri Lanka; 2 Institute of Health Sciences, Universiti Brunei Darussalam, Gadong BE1410, Brunei Darussalam

**Keywords:** feeding preference, malaria, mosquito vectors, sibling species

## Abstract

*Anopheles subpictus* Grassi *s.l.* (Diptera: Culicidae) functions as a secondary malaria vector to *Anopheles culicifacies* Giles *s.l.* (Diptera: Culicidae) in Sri Lanka. The taxon *A. subpictus* is reported to exist as a species complex comprising four sibling species (A–D) that can be differentiated through polytene chromosome banding patterns and stage-specific morphometric traits in India. Based on the morphological characteristics described for the Indian Subpictus Complex, the presence of all four sibling species has been described in Sri Lanka. As sibling species show distinct bio-ecological characteristics that are important for devising appropriate vector control measures, a study was carried out in six districts in the dry zone of Sri Lanka. The results confirm the presence of all four sibling species, with species C predominating in inland areas and species B in coastal areas. Species C and D were indoor-resting and indoor-feeding, while species B was outdoor-resting with no significant preference for indoor- or outdoor-resting. Species B showed distinct morphological variation in the ornamentation of wings and palpi. Blood meal analysis revealed that species B, C, and D can feed on humans as well as cattle. The differential bio-ecological traits shown by the members of the Subpictus Complex are important for developing appropriate vector control measures in Sri Lanka.

## Introduction

**Figure 1.**
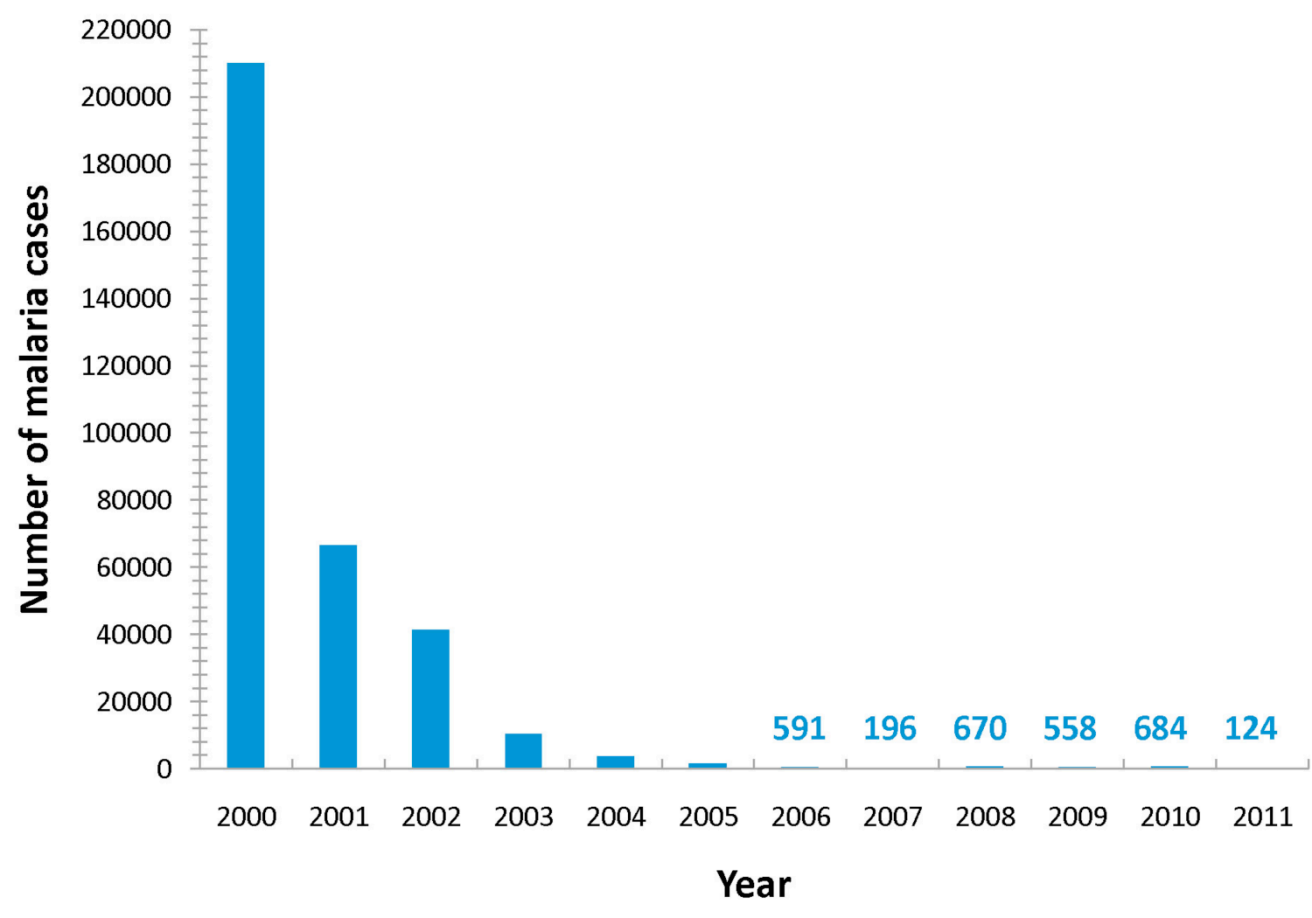
Number of reported malaria cases during 2000–2011 (source: Anti-Malaria Campaign, Sri Lanka).

**Figure 2.**
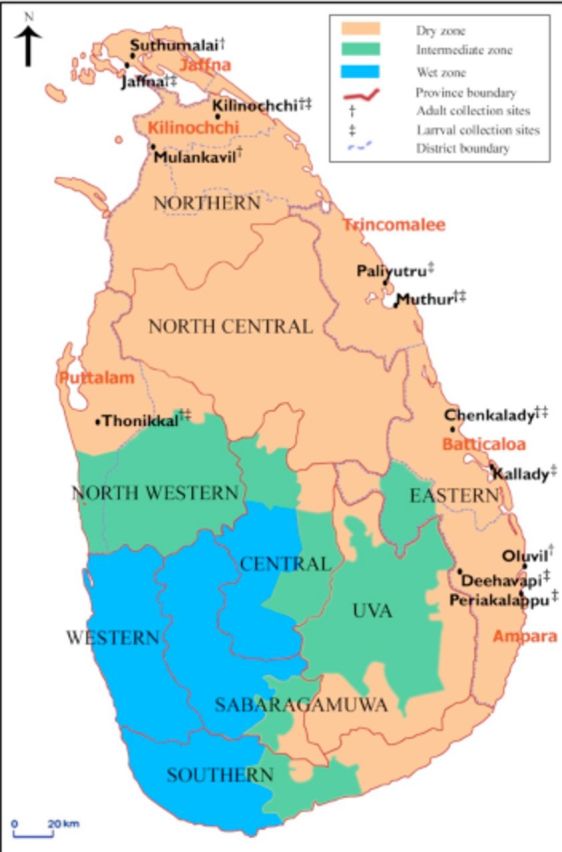
Map showing the location of stu dy sites in the dry zone of Sri Lanka. The island is divided into three clim atic zones based on annual rainfall. The wet zone receives an average annu al rainfall of 250 cm in two main rainy seasons: the southwest monsoo n and the northeast monsoon. The dry zone receives annual rainfall of 60 to 190 cm, mainly during the northeast monsoon. An intermediate zone, with mixed characteristics, lies between the dry and wet zones.

Malaria has been endemic in Sri Lanka for centuries, but there has been a drastic decline in its incidence in recent years ( Figure 1 ) ( Abeyasinghe et al. 2012 ). Malaria, caused mainly by *Plasmodium vivax* and *Plasmodium falciparum* in Sri Lanka, has been endemic in the dry zone of the country due to favorable ecological and climatic conditions ( Figure 2 ). *Anopheles subpictus* Grassi *s.l.* (Diptera: Culicidae) is a well-known vector of malaria in Southeast Asia ( Manguin et al. 2008 ). Although *Anopheles culicifacies* Giles *s.l.* (Diptera: Culicidae) is the most important vector of malaria in Sri Lanka, a subsidiary role for *A. subpictus s.l.* in transmitting malaria in many parts of the country has been reported (Amerasinghe et al. 1992; Surendran and Ramasamy 2010 ). For example, during a peak transmission period, a study in the northern Jaffna district found that *A. subpictus s.l.* had a higher sporozoite rate than *A. culicifacies s.l.* (Thevarasa and Rajendram 1995). The taxon *An. subpictus* is reported to be a complex comprising four sibling species (A–D) that can be differentiated through fixed inversions in the X-arm of the polytene chromosome and stage-specific morphometric characteristics in India ( Suguna et al. 1994 ). An initial study based on a single inversion in the X-arm of the polytene chromosome for Indian species A and B revealed only the presence of species A and B in Sri Lanka ( Abhayawardana et al. 1996 ). Later, studies based on the diagnostic number of egg ridges for Indian sibling species suggested the presence of all four sibling species in the country (Ab-hayawardena et al. 1999). However, a recent study of the internal transcribed spacer 2 (ITS2) and the 28S RNA D3 domain sequences of ribosomal DNA showed that most, if not all, morphologically identified members of *An. subpictus* species B were in fact members of the *Anopheles sundaicus* (L.) complex ( Surendran et al. 2010 , 2013).

The vector control measures in Sri Lanka include indoor residual spraying on a rotational basis, distribution of long-lasting insecticide-treated nets, introduction of larvivorous fish in potential breeding sites, reduction of larval habitats through the filling of abandoned gem pits, and spatial spraying where necessary (Annual Report of Anti-malaria Campaign 2011 ). Members of a species complex show differential susceptibility to insecticides and parasites and differ in their bio-ecological traits ( Subbarao 1998 ). Therefore, the establishment of vector bionomics, such as prevalence and feeding and resting preferences, is important for optimally implementing vector control measures. The present study was carried out to establish prevalence and feeding behavior of the members of the Subpictus Complex in six districts in the dry zone of the country. Considering the genetic similarity of *An. subpictus* species B to *An. sundaicus* in Sri Lanka ( Surendran et al. 2010 ), the morphologically identified species B specimens of the Subpictus Complex are referred as species *B/An. sundaicus s.l.* in this article.

## Materials and Methods

### Adult collection

Adult anopheline mosquitoes were collected monthly between July 2008 and May 2011 from six collection sites ( Figure 2 ). Adult collection techniques, such as cattle baited hut, cattle baited net, pyrethroid spray collection, window trap collection, indoor hand collection, and human landing collection, were employed. The partial monthly outdoor human landing collections involving two human volunteers were carried out from 18:00 to 24:00 hours in Oluvil (7.21° 44.41’ N, 81.50° 42.68’ E) from September 2009 to March 2010.

### Larval collection

Larval forms were also collected between February 2009 and December 2010 from stagnant water bodies associated with inland and coastal areas in the six districts ( Figure 1 ) using a 250-mL capacity dipper, as described by Jude et al. (2010) . Larval collections were carried out in both areas, targeting different breeding sites, ponds, pools, slowly running water, lagoons, boats, wells, rock pools, pits, footprints, and paddy fields. Salinity was measured in the collected water samples with a salinometer (Atago, www.atago.net ).

### Identification of sibling species of the Subpictus Complex

The collected adults and larvae from the Eastern Provincial districts and the Puttalam District were brought to the Zoology laboratory of Eastern University, Chenkalady, Batticaloa, Sri Lanka, and the collected adults and larvae from the Northern Provincial Districts were brought to the Zoology laboratory of the University of Jaffna, Thirunelvely, Jaffna, Sri Lanka. Adult and larval specimens collected by the different techniques were maintained separately and identified as *An. subpictus s.l.* using published keys ( Christophers 1933 ; Amerasinghe 1990 ; Amerasinghe 1992 ). Identified *An. subpictus* larvae were screened for reported characteristics of mesothoracic seta (4M) ( Suguna et al. 1994 ) to identify sibling species. Identified blood-fed females were maintained individually, and single female F1 progeny were raised as described previously ( Surendran et al. 2011 ). Five to 10 eggs from each female were placed on a clean microscopic slide, and the number of ridges on floats was counted under a light microscope (40x, Olympus, www.olympus-global.com ) sibling species were identified based on reported number of egg ridges ( Suguna et al. 1994 ). As morphological variations were noted among mosquitoes identified as species B based on egg morphology, the resultant adult progeny of identified sibling species B were used to study the ornamentation of wings and palpi.

### Blood meal analysis

In order to establish the relative anthropophagic nature of the sibling species’ blood meal, analysis was carried out using antisera to bovine and human blood proteins. Indoor-resting gravid/semi-gravid mosquitoes collected by pyrethroid spray collection and hand collection techniques and identified to sibling species based on morphological characteristics were used for blood-meal analysis. A simple modified precipitation test using micro-capillary technique was used to determine the source of the blood meal ( Tempelis and Lofy 1963 ). The midgut of each identified mosquito was dissected on a clean microscopic slide on a drop of 0.01 M PBS under the dissecting miscroscope (10x, Olympus). The blood meal was diluted in 200 µL 0.01M PBS and taken into the micro Haematocrit tubes (NRIS) with antiserum to human or bovine blood proteins (Sigma-Aldrich, www.sigmaaldrich.com ) separately diluted to 10 ^3^ . The formation of an im-munoprecipation ring at the interface of the two phases (blood and antiserum) was defined as a positive reaction.

### Statistical analysis

A chi-square test was performed to associate feeding and resting preferences sibling species of the Subpictus Complex.

## Results

A total of 9,584 adults and 2,909 larval forms of *An. subpictus s.l.* were collected during the study period ( Table 1 ). The results showed the presences of all four sibling species (A–D) in the collections. Species C (63.7%) was predominant in inland areas, followed by species B (20.8%), D (15%), and A (0.5%). In coastal areas, species B/ *An. sundaicus* s *.l.* was predominant (49%), followed by species C (41%) and species D (10%). Only a few species A were collected during the study period. Statistical analysis revealed a significant association between sibling species and resting (chi-square = 340.7; *P* = 0.000) and feeding (chi-square= 6.0; *P* = 0.01) preferences. Species C (chi-square = 3.9; *P* = 0.04) and species D (chi-square = 421.2; *P* = 000) showed significant preference for indoor resting, while species B/ *An.A. sundaicus s.l.* (chi-square = 536.7; *P* = 0.000) showed significant preference for outdoor resting ( Table 2 ). Significant differences were detected only between species C (chi-square = 505; *P* = 0.01) and D (chi-square = 4.9; *P* = 0.02) in their indoor and outdoor feeding preferences. Species B/ *An. sundaicus s.l.* did not show a significant (chi-square = 0.31; *P* = 0.58) association between their indoor and outdoor feeding preferences.

**Table 1.**
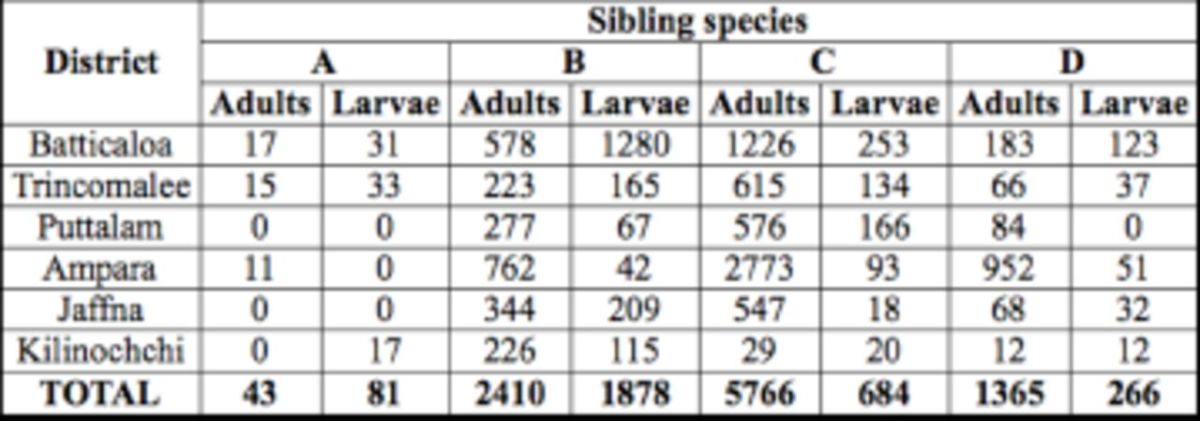
Prevalence of the different sibling species of the Subpictus Complex in adult and larval collections from six districts in the dry zone of Sri Lanka.

**Table 2.**
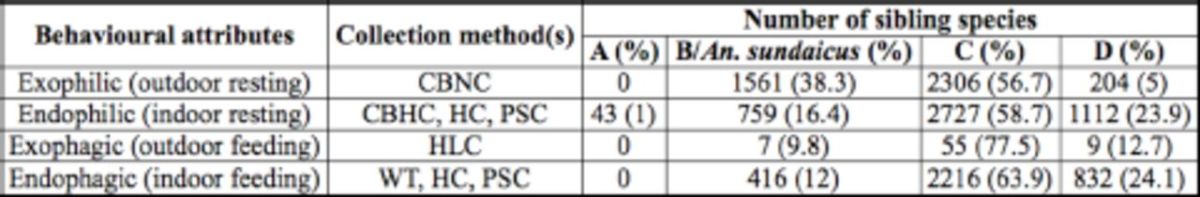
Resting and feeding preferences of sibling species of the Subpictus Complex.

CBHC = cattle baited hut collection, CBNC = cattle baited net collection, PSC = pyrethroid spray collection, WT = window trap collection, HC = indoor hand collection, HLC = human landing collection

**Figure 3.**
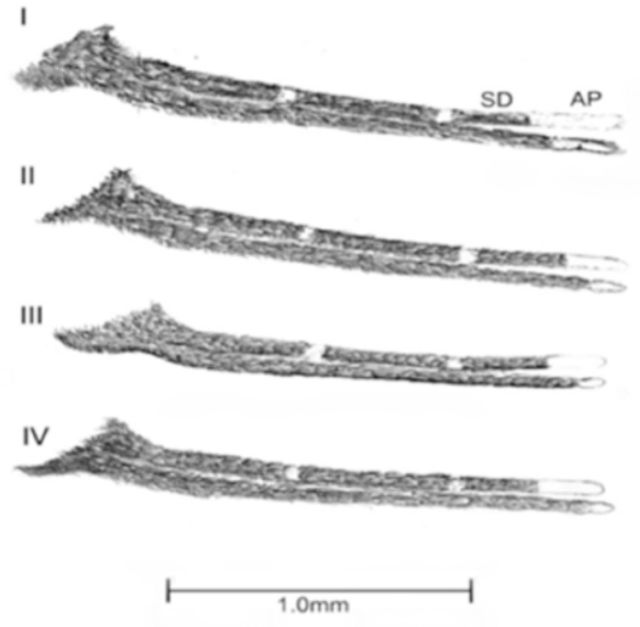
Variations in the ornamentation of palpi of *Anopheles subpictus* species B/ *An. sundaicus s.l* AP - apical pale band. SD - sub-apical dark band.

The adult F _1_ progeny of 135 species B/ *An. sundaicus s.l.* were subjected to morphometric analysis. Based on the ornamentation of wings and palpi, the identified species B/ *An. sundaicus s.l.* could be classified into four categories, termed type I, II, III, and IV. The morphological characteristics for these categories are shown in Figure 3 and listed in Table 3 . Among the four types, type I was the least prevalent, even though it occupied both inland and coastal localities. Type II was prevalent in inland areas, while types III and IV were confined to coastal areas, with Type IV being predominant ( Table 3 ).

**Table 3.**
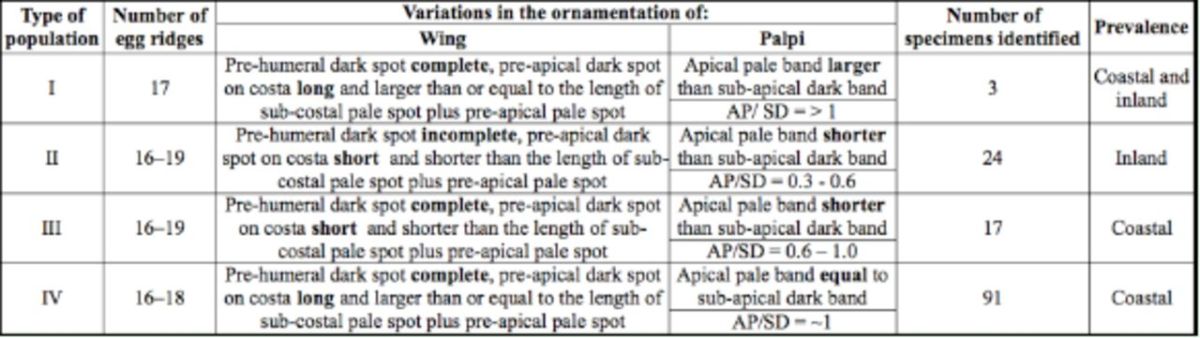
Variations in the ornamentation of wing and palpi among *An. subpictus* species *B/An. sundaicus s.l.*

AP= Apical Pale Band; SD= Sub-Apical Dark Band

A total of 685 blood-fed mosquitoes were tested with antisera to human and bovine blood to characterize the blood meal. These consisted of 14, 126, 220, and 235 species A, B/ *An. sundaicus s.l.* , C, and D, respectively ( Table 4 ). Statistical analysis revealed an association between sibling species and blood meal source (chi-square = 41.61; *P* = 0.000). All sibling species showed both anthropophagic and zoophagic feeding behavior, with the latter significantly predominating in species B (chi-square = 27.5; *P* = 0.000) and D (chi-square = 30.1; *P* = 0.000). Species C did not show any significant preference for blood meal source (chi-square = 4.4; *P* = 0.11). One specimen identified as species B/ *An. sundaicus s.l.* had mixed blood meals (human and bovine). Interestingly, 19% of the blood meal sources could not be identified as either human or bovine. This suggests that the sibling species may also take blood meals from other animal sources.

**Table 4.**
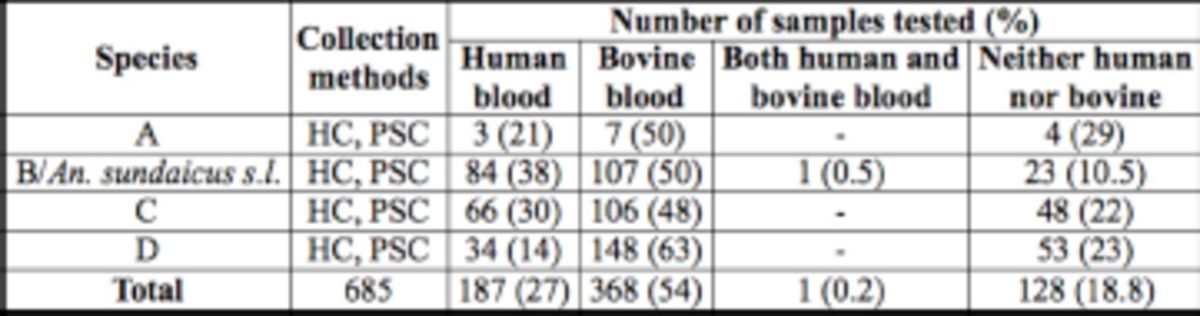
Preference for feeding on human and bovine blood by blood meal analysis

## Discussion

The results suggest that all four sibling species of the Subpictus Complex are widely distributed in the six Sri Lankan districts. Sibling species C was predominant in inland areas and species B/ *An. sundaicus s.l.* in coastal areas. Species A and D also tended to be predominant in inland areas. Different collection techniques revealed that species B */An. sundaicus s.l.* tended to prefer to rest and feed outdoors (exophilic and exophagic), and species C and D preferred indoor resting and feeding (endophilic and endophagic). These behavioral characteristics may lead to a greater insecticide selection pressure on indoor-resting species due to indoor residual spraying with insecticides, which is consistent with a previous observation that species C and D are more resistant to common insecticides than species B */An. sundaicus s.l.* ( Surendran et al. 2012 ).

The sibling species are able to undergo preimaginal development in different habitats, such as ponds, paddy fields, wells, running water, abandoned tanks, and lagoons, in inland and coastal localities. The predominance of larvae of species B/ *An. sundaicus s.l.* in coastal areas is consistent with their greater tolerance of salinity, as reported recently ( Surendran et al. 2011 ; Jude et al.2012 ). Thus, a 15 ppt salinity toler ant test can be useful in separating species B/ *An. sundaicus s.l.* from other sibling species in the country ( Surendran et al. 2011 ). The adaptation to brackish water of species B/ *An. sundaicus s.l.* should be taken into consideration when implementing larval control measures, as coastal populations of *An. subpictus* have been incriminated in malaria transmission in Sri Lanka (Abhayawardana et al. 1996) and India ( Manguin et al. >). Sibling species A, C, and D were generally predominant in inland localities, even though their larvae were found to tolerate salinity levels up to 4 ppt in larval habitats ( Surendran et al.2011 ).

Although variations in the ornamentation of the Subpictus Complex have been reported previously ( Kirti and Kaur 2004 ), this is the first report to show variations among sibling species B/ *An. sundaicus s.l.* of this complex. Members of the species complex are reported to show distinct morphological variations in nearby India ( Suguna et al. 1994 ). However, there should not be any ambiguity or overlapping of species-diagnostic morphological characteristics, as this would result in improper identification and thus could be responsible for failure of control measures if the actual vectors are not correctly identified. Even though the four populations share the same reported range (16-20) in the number of egg ridges ( Suguna et al.1994 ), they show marked variations in morphological characteristics of palpi and wings that are used for identifying anophelines.

It was shown that most, if not all, of the morphologically identified members of species B of the Subpictus Complex are genetically close to *An. sundaicus s.l.* in Sri Lanka ( Surendran et al. 2010 , 2013). This shows that these distinct morphological traits may not correspond to any genetic variation. Hyper-melanic variations due to climatic conditions are also reported to mask identification of morphologically closely related species, as in the case of *Anopheles minimus* Theobald and *Anopheles fluviatilis* James ( Singh et al. 2010 ). Recent molecular characterization of all four morphological types of species B revealed that they are genetically similar, and therefore the observed variations in ornamentation of wings and palpi can be attributed to environmental conditions or ecological niche ( Surendran et al. 2013 )

A recent study from Timor-Leste based on DNA sequences of ITS2 and mitochondrial cytochrome-b revealed that the *An. sundaicus* complex and the *An. subpictus* complex are closely related and further suggested that morphological features that are generally used for species identification could mask the accurate identification of vector species ( Cooper et al. 2010 ). Both the Sundaicus Complex and Subpictus Complex share evolutionary traits in that members of these two complexes are able to breed in brackish waters ( Manguin et al.2008 ). The ambiguity and overlaps in morphological characteristics of the closely related *An. sundaicus, An. subpictus,* and *An-nopheles vagus* Dönitz species from the present and previous reports are summarized in Table 5. Therefore, considering the role of *An. subpictus s.l.* population in malaria transmission, a detailed study of the molecular characterization of these populations is warranted to establish their speciation and phylogenetic relationships with other members of the complex and the closely related vector species *An. sundaicus s.l.* and *An. vagus.* All three species are classified under the same subgenus (Cellia) and series (Pyretoph-orous).

The results showed that all the sibling species were able to feed on both human and bovine blood. There are other mammals (e.g., goats, dogs, deer, and common rodents) that are present in the study areas, but the presence of their blood in the fed mosquitoes was not investigated. This may be responsible for the failure to detect both human and bovine blood in many samples. The human blood index for Species A, *B/An. sundaicus s.l,* C, and D were 21%, 38%, 30%, and 14%, respectively. The human blood index result suggests that species *B/An. sundaicus s.l.* is more anthro-phagic than A, C, and D, and consistent with its transmission of malaria in the coastal areas as in India (Paniker et al *.* 1981). The human landing collection results also indicate anthropophagic behavior of species C, and this species could also play a role in malaria transmission, including in inland areas. Coastal populations of species B (Paniker et al *.* 1981) and inland populations of species A ( Soumendranath and Goutam 2000 ) are reported to be vectors of malaria in India. Previous reports from Sri Lanka suggest the role of coastal (species *B/An. sundaicus s.l.)* ( Abhayawardana et al. 1996 ) and inland populations (likely to be a mixture of A, C, and D) ( Amerasinghe et al. 1992 ) of *the An. subpictus s.l.* in malaria transmission. Sri Lanka had very low prevalence of malaria during the present study period; therefore, the role of each species in malaria transmission could not be established.

As in the case of members of the Culicifacies Complex in Sri Lanka ( Surendran and Ramasamy 2010 ), the members of the Subpictus Complex also show different bio-ecological traits, such as differential feeding and breeding preferences, which are important for devising appropriate vector control measures targeting vector species.
